# Metformin use and mortality and length of stay among hospitalized patients with type 2 diabetes and COVID-19: A multiracial, multiethnic, urban observational study

**DOI:** 10.3389/fendo.2022.1002834

**Published:** 2022-11-09

**Authors:** Emily Miao, Kaleena Zhang, Jianyou Liu, Juan Lin, Donna Yoo, Claudene J. George

**Affiliations:** ^1^ Albert Einstein College of Medicine Bronx, New York, NY, United States; ^2^ Department of Epidemiology and Population Health, Albert Einstein College of Medicine Bronx, New York, NY, United States; ^3^ Montefiore Medical Center, Division of Geriatrics, Albert Einstein College of Medicine Bronx, New York, NY, United States

**Keywords:** diabetes, COVID-19, metformin, urban, multiracial, multiethnic

## Abstract

**Introduction:**

Diabetes mellitus is a common comorbidity among patients with coronavirus disease 2019 (COVID-19). Diabetic patients with COVID-19 have a two-fold increased risk of death and tend to have more severe infection compared to the general population. Metformin, a first-line medication for diabetes management, has anti-inflammatory and immunomodulatory effects. Previous studies focusing on metformin and COVID-19 clinical outcomes have had mixed results, with some showing a mortality benefit or decreased complications with metformin use. To date, few studies have analyzed such outcomes among a diverse, multiracial community.

**Methods:**

This was a retrospective review of patients with Type 2 diabetes and a confirmed COVID-19 infection admitted to an urban academic medical center from January 1, 2020 to May 7, 2020. Baseline characteristics were collected. The primary outcomes of the study were in-hospital mortality and length of stay (LOS).

**Results:**

A total of 4462 patients with Type 2 diabetes and confirmed COVID-19 were identified. 41.3% were Black, and 41.5% were Hispanic. There were 1021 patients in the metformin group and 3441 in the non-metformin group. Of note, more participants in the metformin group had comorbid disease and/or advanced diabetes. We found no statistically significant differences between the metformin and non-metformin group in in-hospital mortality (28.1% vs 25.3%, P=0.08) or length of hospital stay in days (7.3 vs. 7.5, P=0.59), even after matching patients on various factors (29.3% vs. 29.6%, P=0.87; 7.7 vs. 8.1, P=0.23).

**Conclusion:**

While patients had more comorbid disease and advanced diabetes in the metformin group, there were no significant differences with regard to in-hospital mortality or length of stay due to COVID-19 compared to the non-metformin group. Prospective studies are needed to determine if there is clinical benefit for initiating, continuing, or re-initiating metformin in patients hospitalized with COVID-19.

## Introduction

Diabetes mellitus is a common comorbidity among patients with coronavirus disease 2019 (COVID-19). Diabetic patients with COVID-19 have a two-fold increased risk of death and tend to have more severe infection compared to the general population (1, 2). Early case-control studies have shown that COVID-19 infected patients with diabetes had a higher risk of severe pneumonia, as well as elevated tissue injury and inflammatory markers (3, 4). Hyperglycemia is known to interfere with control of viremia and inflammation, predisposing patients to poorer outcomes (3, 5).

The apparent association of glucose metabolism with COVID-19 prognosis calls for a better understanding of anti-diabetic agents that affect blood glucose control. Metformin is a widely used first-line agent for the management of diabetes with anti-inflammatory and immunomodulatory effects (6). It has been shown to decrease TNF alpha and IL-6, which are both proinflammatory adipokines associated with visceral obesity and also implicated in morbidity from COVID-19 infection (6). There have been systematic reviews examining metformin and COVID-19 outcomes (7, 8). Some studies show a mortality benefit (9, 10), while others show no statistically significant association (11, 12). Bramante et al., 2020 found an association of metformin with decreased mortality in women alone, possibly due to sex-specific reduction of TNF alpha (6). Jiang et al., 2020 did not find an effect on 30 day all-cause mortality but found a lower incidence of acute respiratory distress syndrome (ARDS), especially in women (13). A study by Gao et al., 2020 found that metformin users had a higher risk of severe COVID-19 illness, with severity defined in terms of life-threatening complications like ARDS, septic shock, and organ dysfunction requiring ICU admission (14).

In 2010, non-Hispanic Black populations were found to have the highest prevalence of diabetes at 12.6% (15). The racial and ethnic disparities in diabetes morbidity and mortality parallel those observed during the COVID-19 pandemic. A systematic review by Mackey et al., 2020 found with moderate-to-high strength evidence that Black and Hispanic populations experienced higher rates of COVID-19 infection, hospitalization, and mortality compared to non-Hispanic Whites (16). Therefore, it is important to reproduce the studies of COVID-19 and metformin use in a multiracial, multiethnic population. We conducted a retrospective study to compare in-hospital mortality and length-of-stay (LOS) at a major academic center in the Bronx, New York.

## Materials and methods

### Study design and participants

This is a retrospective analysis of patients with type II diabetes and a confirmed COVID-19 infection admitted to an urban academic medical center. COVID-19 was confirmed through polymerase chain reaction (PCR) testing. The study included patients admitted from January 1, 2020 to May 7, 2020 and was approved by the institution’s institutional review board. Baseline characteristics including age, gender, race, ethnicity, comorbidities including hypertension, peripheral artery disease, coronary artery disease, chronic kidney disease, as well as number and type of anti-diabetic medications were collected. Additional data including HbA1c, anion gap at time of admission, creatinine, estimated glomerular filtration rate (eGFR), and body mass index (BMI) were also collected. The primary outcomes of the study were in-hospital mortality and length of stay (LOS).

### Statistical analysis

Statistical analyses were performed using SAS 9.4 (SAS Institute Inc., Cary, NC, USA). Patient demographics and clinical characteristics were summarized for the whole sample and compared between metformin and non-metformin groups using chi-squared or Fisher’s exact tests for categorical variables, and 2-sample t-tests for continuous variables. Univariate analysis was performed to assess the association between in-hospital mortality and length of stay with metformin treatment using chi-squared and 2-sample t-tests, respectively. Propensity score matching was performed on selected demographic and clinical variables by implementing a 1:1 matching algorithm, and the two outcomes were analyzed similarly using the matched data. Differences with a two-sided p-value less than 0.05 were considered to be statistically significant.

## Results

### Patient characteristics

A total of 4462 patients were identified. Patient demographics are shown in **Table 1**. The sample was 46.9% female, with a mean age (SD) of 64.4 (±16.2) years, 41.3% were Black and 41.5% were Hispanic. There were 1021 patients in the metformin group and 3441 in the non-metformin group. The prevalence of co-morbidities was higher in the metformin group when compared to the non-metformin group in such conditions as hypertension (58.2% vs. 37.5%, P<0.0001), coronary artery disease (13.4% vs. 6.7%, P<0.0001), and peripheral artery disease (1.7% vs. 1.0%, P=0.06). BMI (30.45 vs. 29.36, P<0.0001), HbA1c (8.21 vs. 6.52, P<0.0001) creatinine (1.62 vs. 2.14, P<0.0001), anion gap (17.06 vs. 16.62, P<0.01) and mean number of anti-diabetic agents (1.2 vs. 0.22, <0.0001) were also significantly higher in the metformin group. Patients in the metformin group were also significantly more likely to be on concomitant anti-diabetic agents including sulfonylureas (29.5% vs. 3.6%), dipeptidyl-4 inhibitors (36.4% vs. 4.6%), glucagon-like peptides (11.6% vs. 1.7%), sodium-glucose transport-2 inhibitors (7.0% vs. 0.4%), and insulin (36.6% vs. 11.8%) when compared to the non-metformin group (P<0.0001).

**Table 1:** Patient demographics.

	Total (*n* = 4462)	Metformin (*n* = 1021)	No Metformin (*n* = 3441)	p-value
**Age**, mean (SD)	64.38 (16.19)	67.14 (12.47)	63.56 (17.01)	<0.0001
**Gender**, *n* (%)				0.53
Male	2367 (53.1)	533 (52.2)	1834 (53.3)	
Female	2094 (46.9)	488 (47.8)	1606 (46.7)	
**Race**, *n* (%)				<0.01
White	461 (11.3)	89 (9.5)	372 (11.9)
Black	1681 (41.3)	395 (42.1)	1286 (41.0)
Asian	111 (2.7)	39 (4.2)	72 (2.3)
Other	1822 (44.7)	416 (44.3)	1406 (44.8)
**Ethnicity**, *n* (%)				0.33
Hispanic	1659 (41.5)	366 (40.1)	1293 (41.9)
Non-Hispanic	2341 (58.5)	547 (59.9)	1794 (58.1)
**Hypertension**, *n* (%)				<0.0001
Yes	1886 (42.3)	594 (58.2)	1292 (37.5)
No	2576 (57.7)	427 (41.8)	2149 (62.5)
**Peripheral Artery Disease**, *n* (%)				0.06
Yes	50 (1.1)	17 (1.7)	33 (1.0)
No	4412 (98.9)	1004 (98.3)	3408 (99.0)
**Coronary Artery Disease**, *n* (%)				<0.0001
Yes	366 (8.2)	137 (13.4)	229 (6.7)
No	4096 (91.8)	884 (86.6)	3212 (93.3)
**BMI**, mean (SD)	29.62 (7.46)	30.45 (7.67)	29.36 (7.38)	<0.0001
**Chronic Kidney Disease**, *n* (%)				0.71
Yes	313 (7.0)	69 (6.8)	244 (7.1)
No	4149 (93.0)	952 (93.2)	3197 (92.9)
**Sulfonylurea**, *n* (%)				<0.0001
Yes	424 (9.5)	301 (29.5)	123 (3.6)
No	4038 (90.5)	720 (70.5)	3318 (96.4)
**DPP4 Inhibitor**, *n* (%)				<0.0001
Yes	529 (11.9)	372 (36.4)	157 (4.6)
No	3933 (88.1)	649 (63.6)	3284 (95.4)
**GLP-1 Agonist**, *n* (%) Yes	177 (4.0)	118 (11.6)	59 (1.7)	<0.0001
No	4285 (96.0)	903 (88.4)	3382 (98.3)
**SGLT-2 Inhibitor**, *n* (%)				<0.0001
Yes	86 (1.9)	71 (7.0)	15 (0.4)
No	4376 (98.1)	950 (93.0)	3426 (99.6)
**Insulin**, *n* (%)				<0.0001
Yes	780 (17.5)	374 (36.6)	406 (11.8)
No	3682 (82.5)	647 (63.4)	3035 (88.2)
**HbA1c**, mean (SD)	7.02 (2.04)	8.21 (2.12)	6.52 (1.79)	<0.0001
**Anion Gap**, mean (SD)	16.72 (4.68)	17.06 (4.67)	16.62 (4.68)	<0.01
**Creatinine**, mean (SD)	2.02 (2.61)	1.62 (1.67)	2.14 (2.82)	<0.0001
**eGFR**, mean (SD)	56.00 (31.04)	57.37 (28.33)	55.59 (31.82)	0.09
**Number of Medications**, mean (SD)	0.45 (0.85)	1.21 (1.11)	0.22 (0.58)	<0.0001

### Outcomes

Overall, there were no statistically significant differences in in-hospital mortality or length of stay (LOS) between metformin and non-metformin groups. Results from univariate analysis and propensity score matching are shown in **Table 2**. Clinical outcomes of in-hospital mortality (28.1% vs 25.3%, P=0.08) and LOS in days (7.3 vs. 7.5, P=0.59) showed no significant difference between metformin and non-metformin groups. Similar analysis was completed after matching patients in the metformin and non-metformin group on factors noted to be significantly different between the two groups (**Table 1**). Using propensity score matching, participants were matched on factors including age, race, hypertension, coronary artery disease, insulin use, HbA1c, creatinine, BMI, and total number of anti-diabetic medications (not shown). Analysis of matched groups still showed no observable differences in either endpoint of mortality (29.3% vs. 29.6%, P=0.87) or LOS (7.7 vs. 8.1, P=0.23).

**Table 2:** COVID-19 Clinical outcomes and metformin use.

	Metformin(*n* = 1021)	No Metformin(*n* = 3441)	p-value*	Metformin(*n* = 796)	No Metformin(*n* = 796)	p-value**
**In-Hospital Mortality**, *n* (%)			0.08			0.87
Alive	734 (71.9)	2569 (74.7)		563 (70.7)	560 (70.4)	
Deceased	287 (28.1)	872 (25.3)		233 (29.3)	236 (29.6)	
**Length of Stay**, mean (SD)	7.3 (6.7)	7.5 (6.7)	0.59	7.7 (6.8)	8.1 (7.1)	0.23

*Comparison on original data.

**Comparison on PSM data matched on age, race, hypertension, coronary artery disease, insulin use, HbA1c, creatinine, BMI, and total number of anti-diabetic medications.

## Discussion

While metformin may theoretically reduce inflammatory cytokines that are implicated in COVID-19 progression, there are mixed results regarding whether metformin decreases COVID-19 morbidity and mortality in diabetic patients. To our knowledge, this is one of few studies describing metformin use and COVID-19 outcomes in an urban, multiracial, multiethnic community.

We did not observe differences in in-hospital mortality or length of stay between the metformin and non-metformin groups. Additionally, propensity score matching analysis did not show a statistically significant difference in mortality between the groups. Chronic kidney disease (CKD) burden was comparable between groups, indicating that the non-metformin group did not have significant contraindications to being prescribed metformin. We noted a mean HbA1c of 6.52% (SD 1.79) in the non-metformin group, which suggests that some patients may initially have been diagnosed with diabetes, but lowered their HbA1c through successful treatment. Patients at the cutoff point with newly diagnosed diabetes may also have opted for aggressive diet and exercise rather than pharmacologic modalities. Additionally, despite metformin being a first-line agent for the treatment for diabetes, a significant proportion (n=3441) of patients were not on metformin. This could have been due to renal impairment, the intolerability of metformin’s gastrointestinal side effects, patients being lost to outpatient follow-up after the initial diabetes diagnosis, or failure to pick up newly dispensed medications. Furthermore, while other studies have noted mortality benefits in women alone (6), we did not identify such gender-specific differences.

Of note, the metformin and non-metformin groups were imbalanced. The metformin group was more likely to have a higher comorbidity burden including hypertension (HTN), coronary artery disease (CAD), and peripheral artery disease (PAD). They had a higher mean body mass index (BMI), mean HbA1c, and mean number of anti-diabetic agents including insulin. Many of these conditions, including HTN, obesity, and cardiovascular disease have known associations with poorer COVID-19 outcomes (17). In contrast, the non-metformin group had a mean HbA1c close to the lower limit of a diagnosis of diabetes, suggesting that patients early on in the diagnosis or that have been successfully treated are disproportionately represented. Therefore, a mortality benefit from metformin, if any, may have been masked by a metformin group that had far more comorbid disease and advanced diabetes. While difficult to ascertain, metformin may even have played a role in offsetting the severity of COVID-19 disease and, subsequently, preventing patients with diabetes and COVID-19 from being admitted and hospitalized, being treated at home, and thus excluded from this study. Furthermore, it is possible that the metformin group would have had a higher mortality and a longer in-hospital length of stay, but did not due to the benefits of metformin.

There may be evidence that the overall health of the metformin group may be related to a detectable difference in study outcomes. The CORONADO study was a large European observational study (*n* = 2449) that evaluated mortality and disease complications among patients with diabetes hospitalized with COVID-19 (18). In contrast to our study, their metformin group was overall younger and more racially and ethnically homogenous. They had a shorter duration of diabetes, fewer diabetic complications, and lower rates of comorbidities compared to the non-metformin group. They were more often using other oral agents; insulin therapy was two times less prevalent. The CORONADO study found that metformin users had significantly lower mortality rates on day 7 (8.2% vs. 16.1%, P<0.0001) and day 28 (16.0% vs. 28.6%, P<0.0001) compared to the non-metformin population (18).

In comparison, the TOGETHER trial (*n* = 418) is the first known randomized controlled trial to evaluate metformin in reducing time to hospitalization and mortality in an ambulatory setting (19). Symptomatic patients with a positive COVID-19 test were randomized to metformin vs. placebo for 10 days. The patient characteristics of the two groups were generally balanced with respect to age, BMI, and co-morbidities. Ultimately, the study did not find that metformin had a clinical benefit in the outpatient setting, and did not recommend repurposing of metformin as early treatment for COVID-19 disease (19). Further studies can examine metformin in COVID-19 recovered patients to assess when to re-initiate treatment. To evaluate the protective mechanism of metformin, these studies may choose to trend objective signs of disease recovery including inflammatory markers or viral load.

Most studies evaluating metformin use and COVID-19 clinical course have been conducted outside of the United States (U.S.), and fewer have evaluated clinical outcomes among a racially and ethnically diverse patient population (9). To the best of our knowledge, the only other study in the U.S. that has characterized patient demographics and clinical outcomes in an urban cohort was conducted by Crouse et al., 2020 (9). Although the study authors found metformin use to be associated with reduced mortality in patients with COVID-19 and diabetes, their sample size of metformin users was limited (*n* = 76) (9). An international study in the UK by Heald et al., 2022 found social disadvantage, as assessed by the Townsend score, to be associated with increased mortality from COVID-19 independent of diabetes status, and metformin use with decreased mortality (20). The intersectionality of race and economic status, however, calls for further consideration of such variables in a study population and healthcare setting more reflective of those communities with high disease burden in the U.S.

There are several strengths and limitations to this study. Strengths of this study include a large sample size and a focus on a highly diverse, at-risk population that is underrepresented in scientific literature. Given that Black and Hispanic populations experienced higher rates of COVID-19 infection, hospitalization, and mortality compared to non-Hispanic Whites (16), it is important to explore the utility of medications with proposed benefits in these specific populations. Our study builds a foundation for further investigations into the proposed benefits of medications that may decrease mortality in patients with diabetes and COVID-19. Our study adds to the observational work by Crouse et al., 2020, which was also conducted in a diverse urban cohort, but was limited by sample size of their metformin users (*n* = 76) (9). There are several limitations to our study. Due to the retrospective nature of our study, there may have been inconsistencies in provider medication reconciliation, limiting reliability of chart documentation. Information including metformin dose, formulation, adherence, and duration of use were unable to be obtained from chart review. Many variables including smoking status, diabetes duration, and mean blood glucose levels were not collected, which may have influenced the outcomes. For example, smoking is an independent risk factor for mortality in COVID-19 patients and therefore, could have been a confounding variable in our study (21).

## Conclusion

While patients had more comorbid disease and advanced diabetes in the metformin group, there were no significant differences in in-hospital mortality or length of stay due to COVID-19 compared to the non-metformin group. Prospective studies in diverse populations are needed to determine if there is clinical benefit for initiating, continuing, or re-initiating metformin in patients with COVID-19.

## Data availability statement

The de-identified data set is shared by individuals on the institutional IRB and is not publicly available. Requests to access these datasets should be directed to CG: email: clgeorge@montefiore.org.

## Ethics statement

The studies involving human participants were reviewed and approved by Albert Einstein College of Medicine, Montefiore Medical Center Institutional Review Board. Written informed consent for participation was not required for this study in accordance with the national legislation and the institutional requirements.

## Author contributions

EM: Design, data management, interpretation of data, and preparation of manuscript; KZ: Design, data management, interpretation of data, and preparation of manuscript; JiL: Data management, analysis, interpretation of data, preparation of manuscript; JuL: Data management, analysis, interpretation of data, preparation of manuscript; DY: Interpretation of data, preparation of manuscript; CG: Design, acquisition of subjects, data management, analysis, interpretation of data, preparation of manuscript. All authors contributed to the article and approved the submitted version.

## Funding

This work was supported by a National Institute of Health/National Institute of Aging (K23 AG062807-02). The funding source has no role in the study design; in the collection, analysis and interpretation of data; in the writing of the report; and in the decision to submit the article for publication.

## Conflict of interest

The authors declare that the research was conducted in the absence of any commercial or financial relationships that could be construed as a potential conflict of interest.

## Publisher’s note

All claims expressed in this article are solely those of the authors and do not necessarily represent those of their affiliated organizations, or those of the publisher, the editors and the reviewers. Any product that may be evaluated in this article, or claim that may be made by its manufacturer, is not guaranteed or endorsed by the publisher.
